# PD-1/PD-L1 regulates Treg differentiation in pregnancy-induced hypertension

**DOI:** 10.1590/1414-431X20187334

**Published:** 2018-05-28

**Authors:** Lai Jiang, Chaoliang Tang, Yanping Gong, Yujie Liu, Jie Rao, Suyu Chen, Wanjun Qu, Dabao Wu, Lei Lei, Ling Chen

**Affiliations:** 1 Department of Obstetrics and Gynecology, The First Affiliated Hospital, University of Science and Technology of China, Hefei, China; 2 Department of Obstetrics and Gynecology, Anhui Provincial Hospital, Anhui Medical University, Hefei, Anhui Province, China; 3 Department of Anesthesiology, The First Affiliated Hospital, University of Science and Technology of China, Hefei, China; 4 Department of Anesthesiology, Renmin Hospital of Wuhan University, Wuhan, Hubei Province, China

**Keywords:** PD-1/PD-L1, Treg, PIH, Foxp3, Differentiation

## Abstract

Pregnancy-induced hypertension (PIH) causes significant maternal and fetal morbidity and mortality. A decreased number of regulatory T (Treg) cells is associated with the pathogenesis of PIH. The programmed cell death-1 (PD-1)/PD-ligand 1 (PD-L1) pathway is critical to normal pregnancy (NP) by promoting Treg cell development. However, the relationship between PD-1/PD-L1 and Treg differentiation in PIH has not been fully elucidated. In this study, venous blood was obtained from 20 NP and 58 PIH patients. Peripheral blood mononuclear cells (PBMCs) were isolated from venous blood. The levels of Treg-related cytokines (TGF-β, IL-10, and IL-35) in serum and PBMCs were measured by ELISA. The percentage of Treg cells in PBMCs was assessed by flow cytometry. The mRNA levels of Treg-specific transcription factor Foxp3 in PBMCs, and PD-1 and PD-L1 in Treg cells were detected by qRT-PCR. The protein levels of PD-1 and PD-L1 in Treg cells were evaluated by western blot. The serum levels of TGF-β, IL-10, IL-35, and Foxp3 mRNA expression and CD4^+^CD25^+^ Treg cell percentage in PBMCs were decreased in PIH. Furthermore, a significant increase of PD-1 in Treg cells was found in PIH compared with NP. In addition, PD-L1 Fc, an activator of PD-1/PD-L1 pathway, increased Treg cell percentage, enhanced Foxp3 mRNA expression, and elevated levels of TGF-β, IL-10, and IL-35 in PBMCs. However, anti-PD-L1 mAb exerted a reverse effect. These findings revealed that PD-L1 Fc had a favorable effect on Treg cell differentiation, indicating a potential therapeutic value of PD-1/PD-L1 pathway for PIH treatment.

## Introduction

Pregnancy-induced hypertension (PIH) syndrome complicates 6–10% of pregnancies and causes significant maternal and fetal morbidity and mortality (1). PIH is defined as systolic blood pressure >140 mmHg and diastolic blood pressure >90 mmHg (2) and includes hypertensive disorder complicating pregnancy (HDCP) and preeclampsia (PE). PE is a pregnancy-specific disorder that is traditionally diagnosed by the combined presentation of high blood pressure and proteinuria (3). The ambulatory monitoring of 24-h blood pressure seems to have a role in predicting the deterioration from HDCP to PE. Mothers who have undergone a PIH, especially PE, will be under an increased long-term risk of abruptio placentae, cerebrovascular events, and organ failure. Fetuses of these mothers are at greater risk of intrauterine growth retardation, prematurity, and intrauterine death (4). Thus, understanding the etiology of PIH is critical.

There is increasing evidence indicating that PIH is closely correlated with the immune system. Pregnancy is a cooperative interaction between the mother and her fetus, which is semi-allogeneic in relation to the maternal immune system yet is tolerated during normal pregnancy (5). However, fetal alloantigens encoded by polymorphic genes inherited from the father can provoke a maternal immune response leading to fetal rejection, resulting in pregnancy failure and pregnancy complications, such as PIH and recurrent miscarriage (5). CD4^+^ T lymphocytes, the major cell population involved in the cell-mediated immune response, play a crucial role in the development of PIH. Depending on the changes in different cytokine-induced microenvironments, CD4^+^ T lymphocytes can differentiate into T helper type 1 (Th1), Th2, Th17 or CD4^+^CD25^+^ regulatory T (Treg) subsets performing inflammatory, regulatory or suppressor functions (6,7). Treg cells play a critical role in immunoregulation and induction of maternal-fetal immunotolerance during pregnancy (8,9). The master gene for Treg cells differentiation is transcription factor forkhead box P3 (Foxp3). Abnormal function or a decreased number of Treg/Foxp3 cells is associated with pregnancy failure (10,11). Th1/Th2/Th17 and Treg lineages are associated with each other, and they are able to convert to other lineages (8). The balance of Th1/Th2/Th17/Treg paradigm is related to achievement of maternal-fetal immunotolerance and thus is of importance during normal pregnancy (12). Overexpression of Th1/Th2/Th17 or/and suppression of Treg are proposed to be important factors in pregnancy complications including PIH (8). Therefore, reversing the imbalance of Th1/Th2/Th17/Treg may provide insight into therapeutic options for inducing maternal-fetal immunotolerance and preventing PIH.

The programmed cell death-1 (PD-1)/PD-ligand 1 (PD-L1) pathway is critical to immune homeostasis. Recently, the relevance of Treg cells and PD-1/PD-L1 pathway in controlling immune responses has been highlighted. Of note, PD-L1 enhances the stability of Treg cells and promotes Treg cell differentiation (13–15). By promoting Treg cell development and inhibiting effector T (for example, Th17) cell responses, PD-1/PD-L1 pathway has emerged as an important mediator in terminating the immune response and inducing immune tolerance (16). PIH patients showed a decreased percentage of CD4^+^CD25^+^Foxp3^+^Treg cell in the total CD4^+^T cells compared with normal pregnancy (NP), indicating that Treg was implicated in the development and progression of PIH. This provides insight into the mechanism of PIH, providing Treg as a potential target for clinical treatment. However, the direct evidence of the role of PD-1/PD-L1 and the relationship between PD-1/PD-L1 and Treg in PIH requires further research. Accordingly, in this study, we hypothesized that PD-1/PD-L1 might alleviate PIH by regulating Treg differentiation. First, we explored cell quantities and function of Treg in PIH. Then, we investigated the relationship between the PD-1/PD-L1 pathway and Treg differentiation in PIH.

## Material and Methods

### Study population

All study subjects were recruited from the Department of Obstetrics and Gynecology at Anhui Provincial Hospital of Anhui Medical University. This study included 20 normal pregnancies (NP) and 58 pregnant women suffering from PIH syndrome including severe PE (n=20), mild PE (n=18), and HDCP (n=20). There was no significant difference in age and gestational age between groups. The presence of PE was assessed according to ACOG guidelines (ACOG Task Force on Hypertension in Pregnancy, 2013). All pregnancies were singleton gestations, and none of the participants had active labor at the time of enrollment and blood sampling. Women with pre-existing renal diseases, chronic hypertension before pregnancy, diabetes, recurrent miscarriage, disorders of the immune system, or using immune suppressing medication were excluded from this study.

This study was reviewed and approved by the Clinical Trial Ethics Committee of Anhui Provincial Hospital of Anhui Medical University. All procedures were carried out in strict accordance with the approved guidelines and regulations. Written informed consent was obtained from each participant prior to entering the study.

### Blood sample preparation

Blood samples (5 mL) were obtained by venipuncture from severe PE, mild PE, HDCP, and NP women. Two milliliters was used for the preparation of serum, while the remaining 3 mL was heparinized for the isolation of peripheral blood mononuclear cells (PBMCs) by density gradient centrifugation. The serum was separated and stored at −70°C until required for cytokine determination using an enzyme-linked immunosorbent assay (ELISA). To illustrate the effect of the PD-1/PD-L1 pathway on Treg cell differentiation, PD-L1 Fc (an activator of PD-1 pathway, 10 μg/mL, cat. No. 156-B7-01M; R&D Systems) and anti-PD-L1 mAb (10 ng/mL, cat. no. 25-5982-80; eBioscience, USA) were added to the PBMCs from NPs and PIH pregnancies for incubation of 3 days. PBMCs were isolated for flow cytometry, quantitative polymerase chain reaction (qPCR), western blot, and ELISA. All blood samples were obtained before the PE patients received treatments such as steroids or antihypertensive drugs.

### ELISA

The levels of transforming growth factor β (TGF-β), interleukin-10 (IL-10), and IL-35 in serum or cell culture fluid of PBMCs were measured with an ELISA kit (R&D Systems, USA), according to the manufacturer*'*s protocol.

### Treg differentiation

Treg differentiation was performed by flow cytometry. In brief, 2×10^6^ PBMCs were resuspended and stained with anti-CD4-FITC monoclonal antibodies (mAbs) and anti-CD25-PE mAbs for surface antigens (eBioscience) according to the manufacturer's instructions. The cells were then permeabilized with permeabilization/fixation buffer (eBioscience). The cells were resuspended in 300 μL of PBS for subsequent flow cytometric analysis. Data were acquired using a FACScalibur (BD Biosciences, USA), and processed using the CellQuest program (Becton Dickinson, USA).

### Treg cell isolation

Treg cells were isolated from PBMCs of the women by multi-step magnetic sorting using a human CD4^+^CD25^+^ Regulatory T Cell Isolation Kit (Miltenyi Biotec, USA), with a Midi&Mini MACS instrument (Miltenyi Biotec) following the manufacturer*'*s instructions.

### Quantitative real-time PCR (qRT-PCR)

Total RNA was extracted and purified using TRIzol reagent (Invitrogen, USA), and an equal amount of total RNA (1 μg) was used for cDNA synthesis (Takara Bio, Japan). The primer sets used in this study were designed using Primer-BLAST software and were as follows: PD-1 Forward: 5′-ACCCTGGTGGTTGGTGTCGT-3′, Reverse: 5′-CCTGGCTCCTATTGTCCCTC-3′; PD-L1 Forward: 5′-TTTGCTGAACGCCCCATA-3′, Reverse: 5′-TGCTTGTCCAGATGACTTCG-3′; Foxp3 Forward: 5′-CACTGACCAAGGCTTCATCTG-3′, Reverse: 5′-GGAGGAACTCTGGGAATGTG-3′; GAPDH Forward: 5′-GGACCTGACCTGCCGTCTAG-3′, Reverse: 5′-GTAGCCCAGGATGCCCTTGA-3′. The cDNA (2 μL) was subjected to qRT-PCR amplification analysis using SYBR Green PCR mix (Applied Biosystems, USA). The amount of target relative to a calibrator was computed by 2^−ΔΔCT^, and GAPDH was used for normalization.

### Western blot

Protein was isolated from Treg cells that were lysed in radioimmunoprecipitation buffer (RIPA) containing protease inhibitors at 4°C for 30 min. Cell lysates were prepared with a RIPA lysis buffer kit (Santa Cruz Biotechnology, Inc., USA), Treg cell:lysis buffer of 1:2 (v:v), and the protein concentrations were quantified using a Bio-Rad protein assay (Bio-Rad Laboratories, Inc., USA). Subsequently, equal proteins were separated by 10% SDS-PAGE gels and transferred onto PVDF membranes (Millipore, USA). After blocking with 5% fat-free milk, primary antibodies against PD-1 (cat. No. bs-23426R; Bioss, China) and PD-L1 (cat. No. sc-293425; Santa Cruz Biotechnology, Inc.) were added, followed by secondary antibody horseradish peroxidase-conjugated goat anti-rabbit IgG. GAPDH was used as the loading control. The protein was detected with an enhanced chemiluminescence kit (Applygen Technologies, China) and the band intensity was quantified with Image-Pro Plus 6.0 software.

### Statistical analysis

All statistical analyses were performed using SPSS 16.0 (SPSS Inc., USA). The data were analyzed using Student*'*s *t*-test between two groups and reported as means±SD. All experiments were repeated at least three times. P<0.05 was considered statistically significant.

## Results

### Treg-related cytokines were decreased in PIH

**Figure 1.**
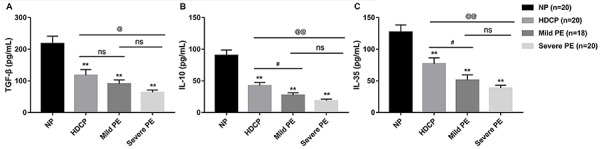
The serum levels of *A*, TGF-β, *B*, IL-10, and *C*, IL-35 in normal pregnancy (NP), hypertensive disorder complicating pregnancy (HDCP), mild preeclampsia (PE), and severe PE were measured with commercial ELISA kits. Data are reported as means±SD. **P<0.01 *vs* NP; ^#^P<0.05 mild PE *vs* HDCP; ^@^P<0.05, ^@@^P<0.01 severe PE *vs* HDCP; ns: not significant (*t*-test).

Treg cells maintain immune homeostasis and tolerance by cell-to-cell contact and by secretion of cytokines (17,18). As shown in Figure 1, significantly decreased serum concentrations of TGF-β, IL-10, and IL-35 were observed in severe PE, mild PE, and HDCP pregnancies compared with NPs. Furthermore, with increased hypertension severity, that is, from HDCP to mild PE and then to severe PE, levels of these cytokines decreased gradually. Moreover, compared with HDCP pregnancies, both severe PE and mild PE patients showed a significant decrease in serum levels of IL-10 and IL-35. As for TGF-β, severe PE patients showed significantly decreased serum TGF-β compared with the HDCP pregnancies, whereas there was no significant difference between the HDCP and mild PE group. Furthermore, these three cytokines did not reach a statistically significant difference between mild and severe PE group.

### Treg and Foxp3 were decreased in PIH

**Figure 2.**
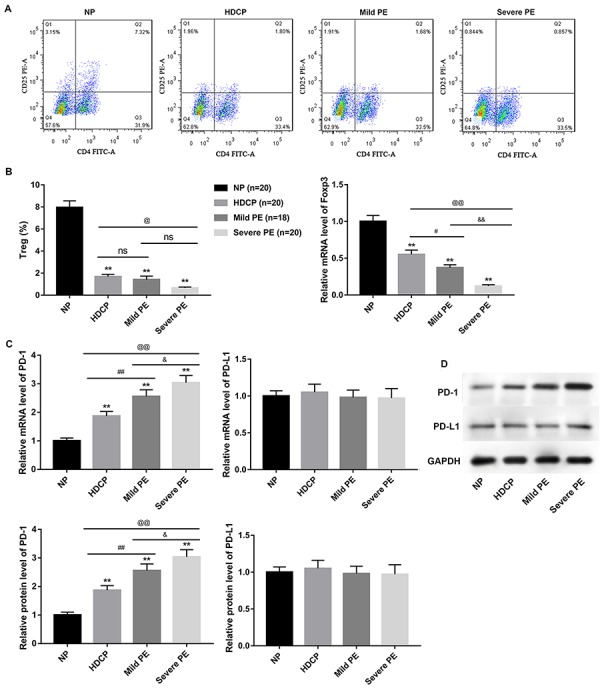
Expression of Treg and Foxp3 as well as PD-1/PD-L1 expression in Treg cells. Peripheral blood mononuclear cells were isolated from the venous blood of women with normal pregnancy (NP), hypertensive disorder complicating pregnancy (HDCP), mild preeclampsia (PE), and severe PE by density gradient centrifugation. *A*, The percentage of Treg cells was assessed by flow cytometry. *B*, The mRNA level of Treg-specific transcript factor Foxp3 was evaluated by qRT-PCR. *C* and *D*, The mRNA and protein levels of PD-1 and PD-L1 in CD4^+^CD25^+^ Treg cells were assessed by qRT-PCR and western blot, respectively. Data are reported as means±SD. **P<0.01 *vs* NP; ^#^P<0.05, ^# #^P<0.01 mild PE *vs* HDCP; ^@^P<0.05, ^@@^P<0.01 severe PE *vs* HDCP; ^&^P<0.05, ^&&^P<0.01 mild PE *vs* severe PE; ns: not significant (*t*-test).

Flow cytometry analysis showed that, similar to the expression pattern of Treg-related cytokines, the percentage of Treg cells in isolated PBMCs of HDCP, mild PE, and severe PE groups was significantly reduced compared with the NP group. Moreover, the percentage of Treg cells in isolated PBMCs of severe PE women was significantly decreased compared with that of HDCP pregnancies (Figure 2A). Furthermore, as shown in Figure 2B, Foxp3 was also significantly decreased at mRNA level in isolated PBMCs of PIH pregnancies compared with that in the NP group. The difference of Foxp3 expression among HDCP, mild PE, and severe PE was significant. Foxp3 is a lineage-specific transcription factor responsible for the differentiation and functions of Treg cells (19). These findings suggested that PIH patients showed a decrease of Treg cells and Foxp3 expression, which might attenuate the immune suppression function of Treg cells.

### Expression of PD-1 and PD-L1 in Treg cells

The mRNA and protein levels of PD-1 and PD-L1 were assessed by qRT-PCR and western blot, respectively. As shown in Figure 2C and 2D, a significant increase of PD-1 in Treg cells was observed in HDCP, mild PE, and severe PE compared with NP, both at mRNA and protein levels. Moreover, with an increased degree of hypertension, levels of PD-1 increased gradually. However, PD-L1 showed no significant difference in isolated PBMCs between NPs and PIH patients. Collectively, these results indicated a potential correlation between the PD-1/PD-L1 pathway and Treg cells in PIH.

### PD-L1 Fc promoted Treg cell differentiation

**Figure 3.**
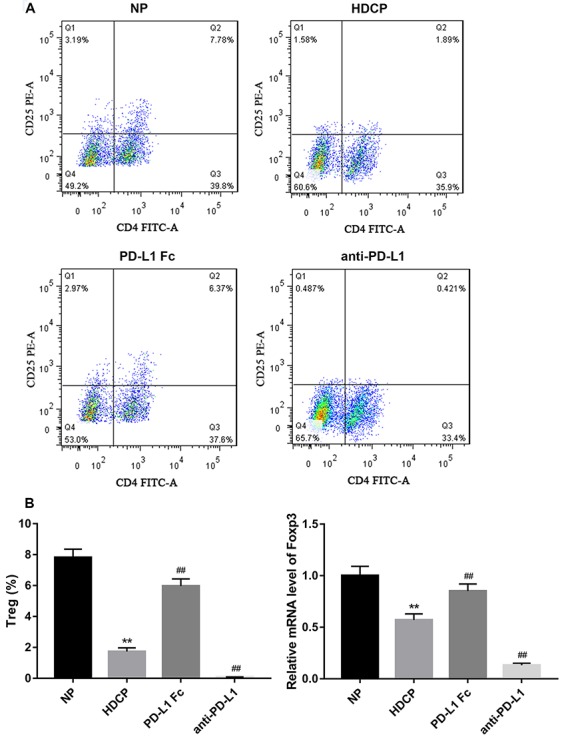
PD-L1 Fc promoted Treg cell differentiation. *A*, PD-L1 Fc (10 μg/mL) and anti-PD-L1 mAb (10 ng/mL) were added into the isolated peripheral blood mononuclear cells (PBMCs) of women with normal pregnancies (NP) and hypertensive disorder complicating pregnancy (HDCP) and incubated for 3 days. *B*, The percentage of Treg cell quantities in PBMCs was evaluated by flow cytometry and the mRNA level of Foxp3 in PBMCs of women with HDCP was evaluated by qRT-PCR. Data are reported as means±SD. **P<0.01 *vs* NP; ^# #^P<0.01 *vs* HDCP (*t*-test).

We found that compared with NP, HDCP showed significantly decreased Treg expression in PBMCs. However, PD-L1 Fc elevated the reduced Treg expression in PBMCs of HDCP. In contrast, anti-PD-L1 mAb further reduced Treg expression in HDCP (Figure 3A). Similarly, the reduced mRNA level of Foxp3 in PBMCs of HDCP was increased by PD-L1 Fc and was decreased by anti-PD-L1 mAb (Figure 3B). These results demonstrated that PD-L1 Fc promoted Treg cell differentiation.

### PD-L1 Fc elevated Treg-related cytokines

**Figure 4.**
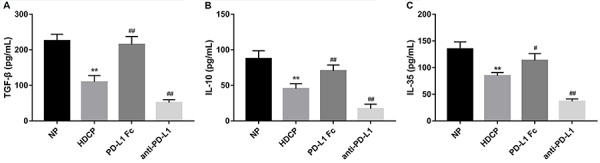
PD-L1 Fc elevated Treg-related cytokines. PD-L1 Fc (10 μg/mL) and anti-PD-L1 mAb (10 ng/mL) were added into the isolated peripheral blood mononuclear cells of women with normal pregnancies (NP) and hypertensive disorder complicating pregnancy (HDCP) and incubated for 3 days. *A*, TGF-β, *B*, IL-10, and *C*, IL-35 levels detected by ELISA. Data are reported as means±SD. **P<0.01 *vs* NP; ^#^P<0.05, ^# #^P<0.01 *vs* HDCP (*t*-test).

In line with the above results, HDCP showed reduced concentration of Treg-related cytokines in cell culture fluid of PBMCs compared with NP. Similarly, these Treg-related cytokines in PBMCs were increased with PD-L1 Fc treatment and decreased with anti-PD-L1 mAb (Figure 4).

## Discussion

Treg cells are a specialized subset of T cells characterized by CD4^+^CD25^+^ cells. With suppressive capacity and regulatory function, Treg cells are major contributors to the tolerance maintenance of the fetus and the maintenance of normal pregnancy (9,20,21). Treg cells have an anti-inflammatory role and maintain tolerance to self-components by contact-dependent suppression or releasing anti-inflammatory cytokine IL-10 and TGF-β (22). TGF-β, IL-10, and IL-35 are cytokines that stimulate the development of adaptive Treg cells (23). Accumulating evidence proved that elevated levels of Treg cells are related to normal pregnancy, whereas deficiencies in their quantity and/or function have been demonstrated in PIH pregnancies (24,25). For instance, absence of Treg cells impairs mice pregnancy, while the adoptive transfer of Treg cells not only rescued pregnancy in abortion-prone mice but also reduced the increased abortion rate in the CBA/J×BALB/c mouse model (26). Consistent with earlier reports, we observed a reduction of these Treg-related cytokines in venous blood, Treg cell percentage, and Treg-specific transcription factor Foxp3 in PBMCs from women suffering from PIH. Therefore, the immune suppression function of Treg cells might be attenuated in PIH. However, the underlying mechanism has not been ascertained.

PD-1 suppresses signaling through the T-cell receptor, resulting in reduced proliferation, cytokine production, and cytotoxic activation of T cells. PD-1 is considered a dominant responsive inhibitory receptor among all of the inhibitory receptors expressed in T cells (27). Apart from autoimmune disorders, the PD-1/PD-L1 pathway also participates in the establishment of maternal-fetal tolerance by promoting the Treg/Th17 balance (28). Engagement of PD-L1 with its ligand, PD-1 on T cells results in the promotion of Treg development and function. Elimination of either Treg cells or PD-1/PD-L1 leads to the breakdown of tolerance and the development of autoimmunity. Despite the reported promotion of Treg cells by PD-1/PD-L1 pathway, the relationship between PD-1/PD-L1 and Treg in PIH requires further investigation. Here, we found increased PD-1 expression in Treg cells from PIH, which was in agreement with the results that higher PD-1^+^ Treg percentage might account for the reduction of Treg cells in PE (29). Thus, we suggest that a dysfunctional PD-1/PD-L1 pathway may account for the decreased Treg cells in PIH patients.

As an activator of PD-1/PD-L1 pathway, PD-L1 Fc has been reported to significantly alleviate symptoms and suppress disease progress in mice suffering from auto-immune disorders by promoting the development of Treg cells (30). PD-L1 Fc can induce a profound increase in the de novo generation of Treg cells from naïve CD4^+^ T cells in the presence of anti-CD3 and TGF-β (13). Furthermore, PD-L1 deficiency led to minimal Treg cell differentiation, highlighting the critical role of PD-L1 during Treg cell differentiation. Therefore, PD-L1 Fc has been considered a rational target for autoimmune disorder therapy. Furthermore, PD-L1 blockade with anti-PD-L1 increased embryo resorption rate and reduced fetus small sizes in early pregnancy (28). As expected, we found that PD-L1 Fc increased Treg cell quantity, enhanced Foxp3 expression by Treg cells, and elevated levels of Treg-associated cytokines. However, anti-PD-L1 mAb exerted a reversed effect. These findings revealed that PD-L1 Fc had a favorable effect on Treg cell differentiation.

As CD4^+^ T lymphocytes can differentiate into the Th1, Th2, Th17 or CD4^+^CD25^+^ Treg subsets (6,7), we used CD4^+^CD25^+^ T lymphocytes to specify Treg cells in this study. Although we have detected mRNA expression of Treg-specific transcription factor Foxp3 in isolated PBMCs of NPs, HDCP, mild PE, and severe PE groups, which could indirectly reflect the expression of Treg cells, other studies have used CD4^+^CD25^+^ Foxp3^+^ T cells to specify Treg subsets. Thus, this might be one of the limitations of this study. Furthermore, recent data showed that CD28 is a major target for PD-1 inhibition both *in vitro* and *in vivo* (31,32). In this regard, it would be interesting to measure CD28 expression in PBMCs, CD4^+^ T cells, or Treg cells in PIH patients compared to NPs. These limitations should be investigated in our future study.

In summary, a reduction of Treg cells was associated with the pathogenesis of PIH. Interestingly, PD-1/PD-L1 pathway promoted Treg cell differentiation in PIH, indicating a potential therapeutic value of PD-L1 Fc for PIH treatment.
